# Evaluation of the Mucoadhesive Properties of Chitosan-Based Microstructured Lipid Carrier (CH-MLC)

**DOI:** 10.3390/pharmaceutics14010170

**Published:** 2022-01-12

**Authors:** Marta Guerini, Giorgia Condrò, Paola Perugini

**Affiliations:** 1 Department of Drug Sciences, University of Pavia, Via Taramelli 12, 27100 Pavia, Italy; giorgia.condro01@universitadipavia.it (G.C.); paola.perugini@unipv.it (P.P.); 2 Etichub, Academic Spin-Off, University of Pavia, Via Taramelli 12, 27100 Pavia, Italy

**Keywords:** Microstructured Lipid Carrier, mucin, cystic fibrosis, chitosan, N-acetylcysteine

## Abstract

Different mucoadhesive systems have been studied in recent years to increase the residence time of the delivery systems and to prolong the release of the drug. The aim of this work was to evaluate the mucoadhesive properties of chitosan-based Microstructured Lipid Carrier (CH-MLC) with a new approach which requires chitosan and mucin to be compacted into a tablet and mucoadhesion to be assessed on a non-mucoadhesive substrate. This type of test showed that chitosan maintains a close bond with mucin even in the presence of a fluid and even encapsulated in microparticles. After this, using a bioreactor, the release of N-acetylcysteine (NAC) from the microparticles (NA-CH-MLC) through a layer of mucus mimicking the pathological conditions of a patient with cystic fibrosis was tested. The release of the active from NAC-CH-MLC demonstrated how the chitosan inside the microparticles acts as a penetration enhancer and how the microparticles can impart a prolonged release over time.

## 1. Introduction

Over the last decades, the development of new dosage forms able to give several advantages over conventional systems already on the market, by introducing mucoadhesive properties and thus enhancing active’s bioavailability, has grown. Therefore, first-pass effect and an early enzymatic or acid degradation of the active substance can be avoided by increasing the residence time in the site of absorption and, hence, ensuring a faster onset of the drug effect [1]. Bio-adhesion can be a suitable mechanism with which it is possible to enhance residence time, especially for drug delivery systems.

Specifically, the mucoadhesive bond of a polymer, as an example of adhesive substance, with mucus, is due to polymer’s interaction with mucins, the main components of mucus. Mucus of a healthy individual consists of mucins, lipids, proteins, salts, macromolecules, cells, bacteria, and water, which represents the 90–98% of mucus weight. Mucins are natural glycoproteins secreted by goblet cells and submucosal glands [2]. These proteins are made up of an amino acid primary sequence that is rich in proline, threonine, and serine, called PTS domains. These amino acids can link with several saccharides, such as galactose, N-acetyl-galactosamine and N-acetylglucosamine, and with fucose or sialic acid [3]. Due to the presence of sialic acids and other negative polar groups (ester sulphates), mucins are negatively charged at pH > 2.6. Moreover, polysaccharides groups form hydrogen bonds with other mucin molecules that, along with disulfide bonds between cysteine residues and hydrophobic interactions with nonpolar groups, contribute to form protein agglomerates varying in weight, from 500 kDa to 20 MDa [4]. Mucoadhesion process can be divided in three main steps: Step I is swelling. Thanks to wetting properties and chain flexibility of the polymer, the polymer itself swells once placed on the mucus membrane, incorporating water, and increasing its size. Step II consists of interpenetration of the polymer with glycoprotein chains on the mucosal layer surface. Step III consists of chemical consolidation [5]. Chemical bonds can be strong, depending on the nature of the force involved.

It is important to understand how the drug and mucus interact and, even more important, how these interactions may affect drug absorption and activity. This consideration includes understanding the diffusion coefficient of the drug through mucus even in disease states. Studies of particle transport through mucus have all shown that it is controlled by the mucus layer, as well as the size, charge, and wettability of the particle surface. The intimacy of contact between a bio-adhesive polymer and biological tissue is best when the surface of the biological tissue is rough. The surface roughness, defined by the aspect ratio (d/h) of the maximum depth (d) to the maximum width (h), must be greater than 1/20 for good adhesion to occur [6].

For surfaces with lower aspect ratios, the viscosity and wetting power of the bio-adhesive become the most important factors required for satisfactory bio-adhesion [6].

The tensile test has been widely used to assess the mucoadhesive power of a wide variety of dosage forms. Ponchet et al., in their work [7], analyzed the tensile force required to separate a mucoadhesive tablet from animal mucin. This force was then used to calculate the work of adhesion. This parameter is calculated as the integration of the force vs. displacement curve obtained in the pulling exercises and has been used extensively over the years as an indicator of the mucoadhesive properties of a material.

Quality by Design (QbD) is defined as “a systematic approach to pharmaceutical development that starts with pre-defined objectives and emphasizes product and process understanding and control, based on sound science and quality risk management” [8]. According to this concept, method quality should be achieved during analytical development. Usually, there are many input factors that can influence the quality of the method. Recently, the Design of Experiments (DoE) has been widely used to understand the effects of multidimensional interactions and input factors on the output responses of analytical methods, as well as actual drug products.

In the DoE approach, input factors are varied to determine their effects on responses (outputs), allowing for the selection of the most important inputs; the identification of the setting of input factors, leading to optimized outputs; and the understanding of the interactions between input factors.

The various types of DoEs can be divided into two macro-areas to better understand their application: (a) screening designs and (b) optimization designs [9,10].

In this specific work, a series of DoEs was chosen precisely for the optimization of a system.

Chitosan is a cationic polysaccharide polymer obtained from chitin by alkaline deacetylation. It consists of N-acetyl-D-glucosamine and D-glucosamine repeating units, linked by (1,4)-β-glycosidic linkage. Due to its biodegradability, biocompatibility and low toxicity, chitosan is widely used inside mucoadhesion forms [11]. Chitosan has –OH and –NH_2_ groups, leading to the capability of forming hydrogen and covalent bonds that play an important role in the mucoadhesion of chitosan [12]. Its structure is rich in NH_2_ groups, with a positive charge at pH < 6.5 (pKa 6.5). This cationic nature provides strong electrostatic interaction with negatively charged components of mucus, including sialic acid, as well as epithelial surfaces.

Cystic fibrosis (CF) lung is characterized by a large amount of sputum that covers the entire surface of the epithelial cells and impairs the mechanical clearance mechanism [13]. Bacterial colonization is promoted by the unique composition of sputum, which contains mucin, lipids, proteins, amino acids, ions, and DNA released by neutrophils in above-average concentrations of a healthy individual [14]. In this environment, microcolonies are formed under anaerobic conditions by adhering to each other and to mucus components, but not to a surface. The microparticle delivery system proposed in this work and already technologically characterized was developed to exert a topic action on the biofilm formation in lungs of patients affected by cystic fibrosis [15,16].

For this reason, the aim of this work was the evaluation of the mucoadhesive properties of N-acetylcysteine (NAC) loaded Microstructured Chitosan–Lipid Carrier (NAC-CH-MLC), and the evaluation of the drug release through a mucus layer.

For this purpose, the first step of the work was to evaluate the interaction between chitosan and mucin at a molecular level by means of a turbidimetric assay. Thereafter, an innovative protocol based on the measurements of the mechanical forces involved in the mucoadhesion process was set up to assess the mucoadhesive capability of microparticles containing chitosan. Two different types of the Design of Experiments were applied to reach these aims.

To test the release profile of NAC from microparticles, an artificial mucus (AM) similar to what pathogenic bacteria produce in the lung, was prepared based on the work of Kirchner S. et al. [17], but suitably modified. Tests were carried out by using an advanced system of cell cultures to perform dynamic 3D models in vitro [18].

## 2. Materials

Chitosan 90/100/A1 MW 86,000 g/mol, deacetylation degree 88–95%; Chitosan 90/400/A1 MW 300,000 g/mol, deacetylation degree 88–95%; and Chitosan 90/2000/A1 MW 1,323,000 g/mol, deacetylation degree 88–95%, were obtained by Kraeber & Co. GMBH, (Ellerberk, Germany). Mucin from porcine stomach Type II and Trehalose, diethylenetriaminepentaacetic acid (DTPA), sterile egg yolk and DNA (Sigma-Aldrich, Milan, Italy); sodium acetate trihydrate (Merck, Milan, Italy); acetic acid 96%, sodium chloride, magnesium stearate and Isooctane (Carlo Erba, Rodano, Italy); *Caesalpinia spinosa* gum (Solagum Tara™, Seppic, Milan, Italy); and glycerol dibehenate (Compritol 888 pastilles, Gattefoss, Milan, Italy) were obtained. Kolliphor P 407, a mix of polyoxyethylene (73%) and polyoxypropylene (POE–POP), was obtained from BASF (Ludwigshafen, Germany). Polyglyceryl-4 sorbitan olivate phosphate (Caldic, Milano, Italy), essential and non-essential L- amino acids (A.C.E.F., Piacenza, Italy) and Tryptone Soya Broth (Oxoid, Basingstoke, UK) were also obtained.

## 3. Methods

### 3.1. Quantitative Evaluation of Mucin–Chitosan Interaction

Mucoadhesion measurements were carried out by means of the turbidimetric assay, using a Spectrophotometer model UV-1900 (Shimadzu, Milan, Italy) and operating at λ = 500 nm [19]. Mucin from porcine stomach type II was dispersed in acetate buffer pH 4.5 at the following concentrations: 0.5, 0.6, 0.8, 0.9 and 1 mg/mL. It was then sonicated for 15 min and filtered with cellulose filter paper (Filter-Lab 1320, 110 mm Ø, FILTROS ANOIA, S. A. Barcelona, Spain). Thus, a calibration curve based on these increasing concentrations was built up. Different types of chitosan solutions in acetate buffer (pH 4.5) were prepared and mixed in a different ratio (*w*/*w*) to mucin. These mixtures were manually stirred and then placed in a thermomixer (Thermomixer comfort-Eppendorf) at 600 rpm for 45 min and 25 °C. Turbidity values were calculated according to the He et al. method: the effective absorbance (A) of the chitosan–mucin mixtures was compared to the theoretical absorbance (A_theor_) calculated by adding the individual absorbance values of the mucin and chitosan solutions in acetate buffer. The difference (Δ Abs = A − A_theor_) was taken as a measure of the interaction between mucin and the chitosan. If Δ Abs ≤ 0 no interaction occurs, if Δ Abs > 0, a strong interaction between mucin and the chitosan is supposed.

#### Design of Experiment I (DoE I)

To investigate the influence of chitosan molecular weight and of the weight ratio between mucin and chitosan on mucoadhesive properties, a 3^2^ model full factorial design was set up by using the Minitab 17^®^ software.

Table 1 reports the independent variables (inputs) and their chosen levels. The response (dependent variable, output) chosen as indicator of the mucoadhesive capability of chitosan is the turbidity value, evaluated with turbidimetric assay, as above reported. The analyses are repeated in triplicate, so the model requires 27 total experiments to be performed.

### 3.2. Evaluation of Mucoadhesion Capability of Chitosan Based Microparticles (CH-MLC)

#### 3.2.1. Tensile-Test Procedure

**Figure 1:**
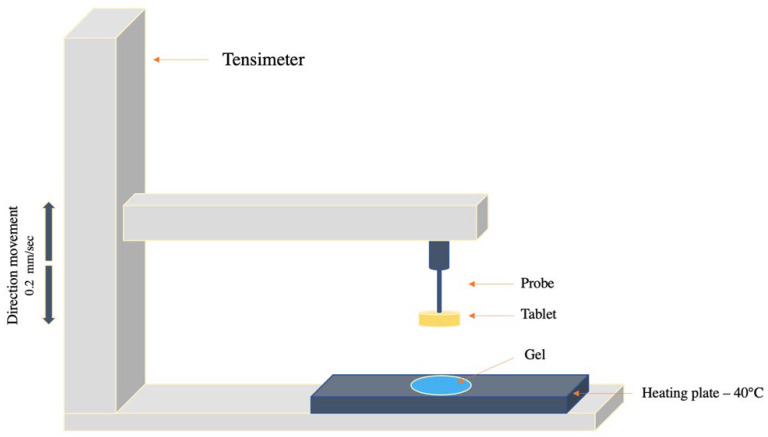
Schematic representation of experiment used for the tensile test.

To quantify mucoadhesion properties, a tensile method with an apparatus model Autograph AGS-D500 (Shimadzu, Milan, Italy) was used. First, 200 mg tablets made of mucin or of a mixture of mucin/microparticles, with several CH-MLC:mucin *w*/*w*, were prepared by a direct compression through a hydraulic press (25 tons Ring Press 00-25 Riic). The tablets obtained have standard dimensions of 2 mm thick, 18 mm in diameter and a surface area of 254 mm^2^. The test procedure involved a preliminary hydration phase in which the entire surface of the tablet was hydrated with 0.9% NaCl; the excess water was afterwards removed. The tablet was then hung on the arm of the instrument, as shown in Figure 1. A total of 5 g of an established gel was placed on a plate and maintained at 40 °C. The tablet was lowered on the surface of the gel and left in contact for 30 s. Detachment analysis was performed at 0.2 mm/sec, until the mucin tablet had completely detached from the gel. For each tablet, analyses were carried out in triplicate, and the results were expressed as maximal strength (mN) and breaking work (mN*mm) required to detach the tablet from the gel.

#### 3.2.2. Tablet’s Surface Energy Measurement

The surface energy of all types of tablets produced was calculated from the measurements of their wettability, using Contact Angle Meter DMe-211Plus (KYOWA Interface Science Co., Ltd., Nobitome, Saitama 352-0011, Japan). The contact angle was used as a measure of the wettability of a liquid on a solid surface. The surface tension of a liquid is given by the sum of its polar component (γ^p^) and its dispersed component (γ^d^), and the same is true for the surface energy of a solid. To calculate the surface energy of the tablets, their contact angle with two solvents, namely one polar and one hydrophobic, was measured. Distilled water (γ_l_^p^ = 50.2 mN/m; γ_l_^d^ = 22.6 mN/m) and isooctane (γ_l_^d^ = 18.7 mN/m) were chosen as polar and hydrophobic solvent, respectively. For isooctane, the polar contribution of surface tension was approximated to 0, being a hydrocarbon chain without polar groups or able to give hydrogen bonds. With the contact angle values obtained with the two different liquids, the surface energy was calculated for each tablet by using the Wu equation [20]:

(1) $$\left(1+\cos\cos\;\theta \right)\gamma_{l}=\frac{4\left(\gamma_{s}^{d}\gamma_{l}^{d}\right)}{\gamma_{s}^{d}+\gamma_{l}^{d}}+\frac{4\left(\gamma_{s}^{p}\gamma_{l}^{p}\right)}{\gamma_{s}^{p}+\gamma_{l}^{p}}$$

θ = contact angle (deg);
γ_l_ = surface tension of the liquid;
γ_l_^d^ = disperse component of the liquid;
γ_l_^p^ = polar component of the liquid;
γ_s_^d^ = disperse component of the solid;
γ_l_^p^ = polar component of the solid.

Cohesion work (w_c_) of particles was calculated with the following formula:

(2) $$w_{c}=2\times \gamma_{s}$$

#### 3.2.3. Design of Experiments II (DoE II)

Before testing chitosan–lipid microparticles, a full factorial design 2^3^ (two levels and three factors) was developed only on mucin tablets in order to optimize the conditions of tablet production. The DoE was applied and analyzed by using the Minitab 17^®^ software. Compression time (20–40 s), compression force (2–10 tons) and hydration time (0–10 min) of the tablets were chosen as independent variables, as pictured in Table 2.

Analyses were performed by using a compound gel to set the process conditions. The gel used in this first phase was made of hyaluronic acid 2%. The surface-energy values, the maximal strength and the breaking work were chosen as responses (output). The analyses were carried out in triplicate, so the model required 24 total experiments to be performed. The samples were also made in a randomized order. Analysis of variance was performed to assess the suitable response surface model and its significance.

Table 3 summarizes all types of mucin tablets produced according to the 2^3^ full factorial design.

#### 3.2.4. Mucoadhesion Test Applied on Chitosan-Based Microparticles (CH-MLCs)

Micro-structured lipid carriers with or without NAC were prepared by a high shear homogenization method, as reported in a previous work [15]. Briefly, MLCs were obtained through a double-emulsion process (W/O/W). The inner phase containing chitosan, and eventually NAC, dissolved in acidic medium was vigorously dripped into the oil phase containing the lipidic component of the particle matrix. This water-in-oil emulsion was then emulsified to the outer aqueous phase to obtain a double emulsion. The solidification of microparticles was achieved in a cold bath. After purification by using the gradient centrifugation (SL 8R, Thermo Scientific, Monza, Italy) method and freeze-drying (Edwards, Cinquepascal, Milan, Italy), CH-MLC and NAC-CH-MLC were then stored as a powder. Different types of tablets (lactose, lactose:mucin 40:60 (*w*/*w*), lactose:mucin 20:80 (*w*/*w*); CH-MLC:mucin 40:60 (*w*/*w*); CH-MLC:mucin 20:80 (*w*/*w*)) were prepared by following the optimized process conditions, and they were analyzed to evaluate the mucoadhesion of chitosan within microparticles. In this study, both mucoadhesive molecules, namely chitosan and mucin, were in the tablet matrix, and the tensile test was performed by using a non-mucoadhesive gel made of Caesalpine gum. Each tablet was made in triplicate, and for each type of tablet, we evaluated the surface energy, the maximal strength, and the breaking work. Moreover, to evaluate the behavior of free chitosan—meaning, not included in a pharmaceutical form—tablets containing the main components of the microparticles (polymer, surfactant, lipids and cryoprotectant) in a physical mixture and mucin (40:60 *w*/*w*) were analyzed in triplicate.

### 3.3. Drug Release from NAC-CH-MLC through Artificial Mucus (AM)

**Figure 2:**
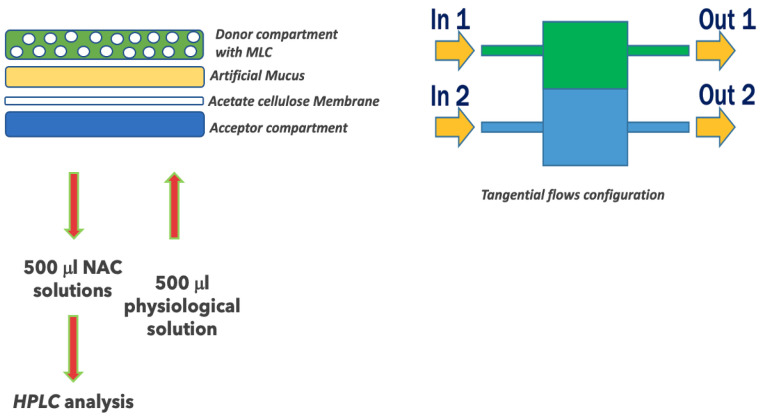
Schematic protocol to prepare LB2 to mimic the physiological barriers of the mucus under dynamic conditions.

An advanced reactor system was used to perform in vitro NAC release from MLC (IVTech ^®^, Lucca, Italy) [18,21]. The system consists of a peristaltic pump, which creates the flow, connected with modular and transparent double-flow bioreactors named LiveBox2 (LB2). LB2 is a dual-flow IVTech bioreactor formed by two chambers, upper and lower, developed to mimic physiological barriers in vitro. LB2 consists of three parts (Figure 2): (1) an apical chamber with a wet volume of 1.5 mL, equipped with an inlet and an outlet tube; (2) a basal chamber with a wet volume of 1 mL, equipped with an inlet and an outlet tube; and (3) a membrane holder placed between the two chambers. The clamp system supplied with the LB2 ensures the watertight closure of the system in both static and dynamic conditions.

An appropriate amount of artificial mucus (AM) was placed on a membrane of cellulose acetate 0.45 μm (previously positioned inside the LB2) to completely cover the surface.

Then a weighted quantity of the freeze-dried microparticles (NAC-CH-MLCs) was placed on the AM. Saline solution (NaCl 0.9%), in the supplied 25 mL plastic bottle, was connected to the pump and to the LB2 by silicon tubes. The flow rate was set up at 0.5 mL/min in the basal compartment.

At fixed times (1, 2, 4, 6 and 8 h), 500 μL was picked up from the acceptor compartment and immediately replaced. The solution withdrawn containing NAC permeated through the mucus was then filtered and analyzed by the HPLC method, already standardized. Briefly, a solution 10:90 (*w*/*w*) of acetonitrile and Milli-Q water at pH 2.5 was used as the mobile phase, and the water Spherisorb 5 μm ODS1 4.6 × 150 mm was used as the column chosen; the flow rate was 0.8 mL/min, and monitoring wavelength was 210 nm.

Six analyses were carried out for each batch. NAC release from MLC was then compared with the passage of NAC powder through the AM, as control.

## 4. Results

### 4.1. Quantitative Evaluation of Mucin–Chitosan Interaction

**Figure 3:**
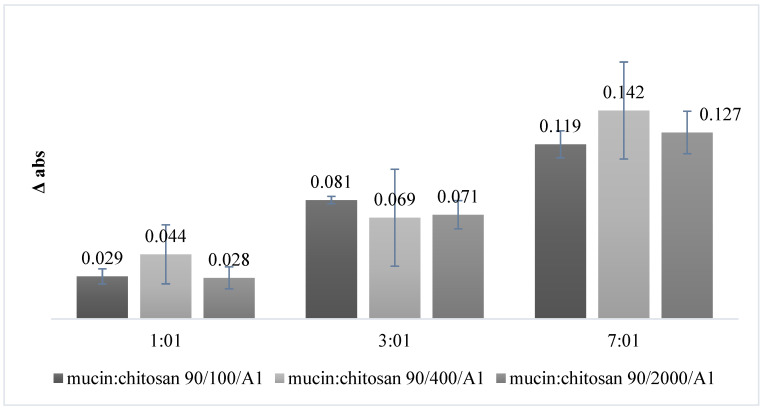
Change in absorbance of different mixtures of mucin:chitosan (*w*/*w*) (01:01, 03:01 and 07:01 *w*/*w*), using three different types of chitosan (90/100/A1; 90/400/A1; 90/2000/A1).

The capability of chitosan to react with mucin was evaluated through a turbidimetric assay. The buildup of a calibration curve with increasing concentrations of mucin made it possible to verify that the absorbance follows a concentration-dependent trend: as the mucin concentration increases, the measured absorbance value increases as well. The correlation coefficient of the curve (R^2^ = 0.999) indicated that data are well correlated. Samples prepared according to the DoE were then analyzed. Figure 3 summarizes the mean absorbance-change values (Δ Abs) obtained from turbidimetric assay. It is possible to notice that the values of Δ Abs are all positive, highlighting that mucin and chitosan in solution interact with each other.

**Figure 4:**
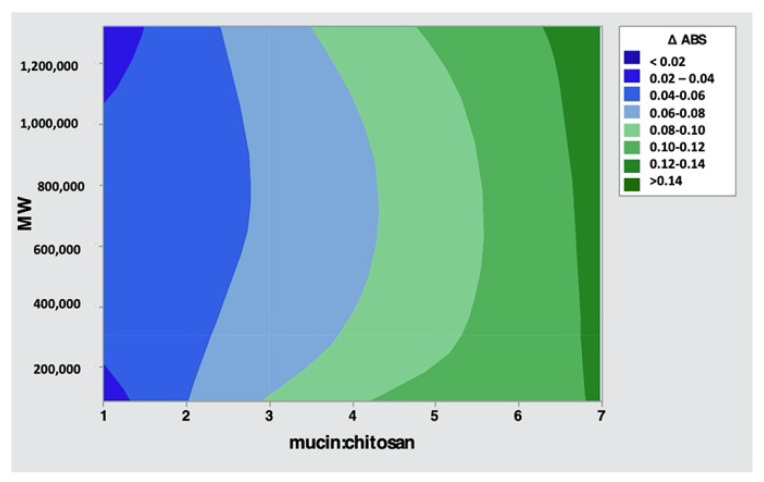
Contour plot of Δ Abs.

Table 4 reports the DoE I analysis of variance. The *p*-value is a probability that measures the evidence against the null hypothesis. Lower probabilities (*p*-value < 0.05) provide stronger evidence against the null hypothesis. Regarding the variance analysis, only the mucin:chitosan factor has a statistically significant effect (*p*-value ≤ 0.05) on the response, and it is also possible to see that no interactions occur between the two factors (*p*-value = 0.592). In order to understand how the factors are connected to each other, the contour plots showing two predictors for the chart are used and reported in Figure 4. The contour plot provides a two-dimensional view in which all points having the same responses are connected

From the contour plot shown in Figure 4, it is confirmed that the ratio (*w*/*w*) between mucin and chitosan of 07:01 permits the stronger bond between mucin and chitosan and that the molecular weight of chitosan does not affect mucoadhesive capabilities.

### 4.2. Tensile-Test Setup

#### Tablet’s Surface-Energy Measurement

The ability of a liquid to spread over is defined as a function of the surface energy of a solid (γ_s_): the higher the surface energy, the greater the ability of the liquid to adhere to it. Furthermore, high surface energy corresponds to high wettability and low contact angle values. For this reason, following the hydration phase, mucin tablets increase the wettability. Table 5 reports the surface energy (γ_s_) and cohesion work (w_c_) values of mucin tablets hydrated and not hydrated. Among the not-hydrated tablets, batch A01 has the greatest surface energy (43 ± 2 mN/m); after hydration, batches B01 and D01 show the higher values, being 69 ± 4 mN/m and 68 ± 4 mN/m, respectively. These last, in fact, were compressed for only 20 s, as reported in Table 3, so they present a greater flexibility that allows the movement of hydrophilic residues on the surface in contact with water.

The cohesion work (w_c_) between mucin particles of the tablets was also calculated. Among the not-hydrated tablets, it is possible to notice that A01s have the greatest cohesion work (87 ± 4 mN × m). In fact, having been compressed for a longer time and with maximum force, particles are in close contact with each other. Among the hydrated particles, it is possible to see that the presence of water increases the cohesion work between the particles in all tablets, particularly B01s and D01s (138 ± 8 mN × m and 138 ± 9 mN × m).

**Figure 5:**
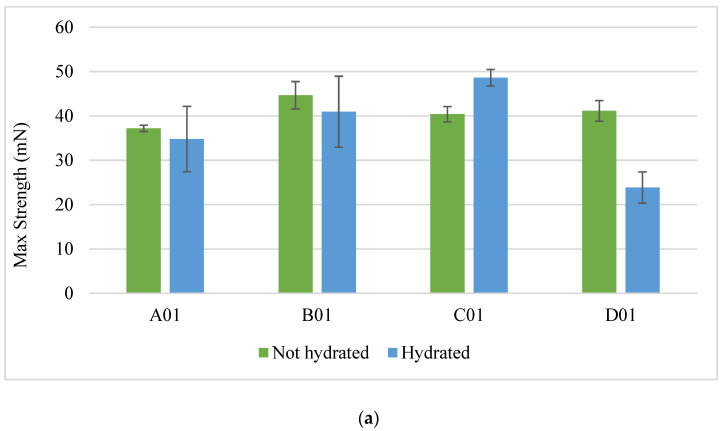

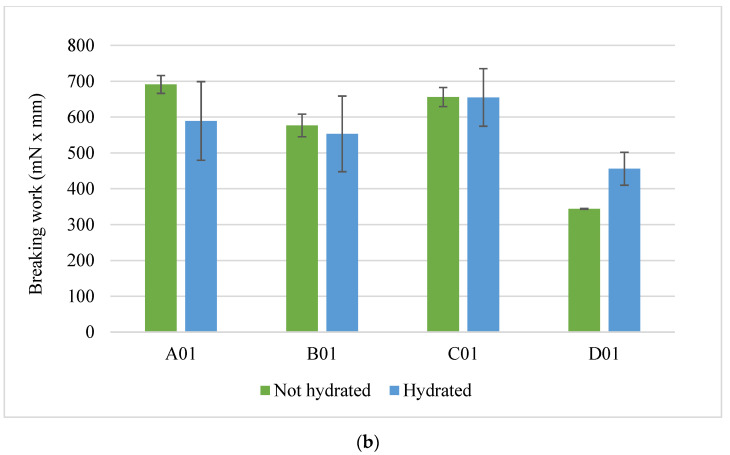
Maximal strength (**a**) and breaking work (**b**) of hydrated and not-hydrated mucin tablets.

The results concerning mechanical tests carried out on hydrated and not-hydrated mucin tablets are reported in Figure 5a,b. Among the not-hydrated tablets, B01 required the greatest strength to detach the tablet from the gel (44.7 ± 3 mN), as shown in Figure 5a; meanwhile, among the hydrated ones, B01 (41 ± 8 mN) and C01 (49 ± 1.8 mN) stand out. By comparing both the average values and the standard deviations of the maximum strength relative to the hydrated and not-hydrated tablets, hydration led to an increase in the releasing force, except for the D01 tablets, where the strength decreases.

Tablets that require more work to break the bond between tablet and gel are A01 and C01, both hydrated and not-hydrated (Figure 5b).

Table 6 shows the variance analysis for each of the three responses: Factors and interactions with a *p*-value ≤ 0.005 statistically affect the response. Compression time and compression force affect the breaking work and surface energy, while their interaction influences the maximal strength and the breaking work. The surface energy is the only answer that is affected by the hydration time. The two-level interaction of hydration time–compression time influences the maximal strength, while the hydration time–compression force interaction affects the surface energy. Finally, the three-level interaction impacts the maximal strength and the surface energy.

**Figure 6:**
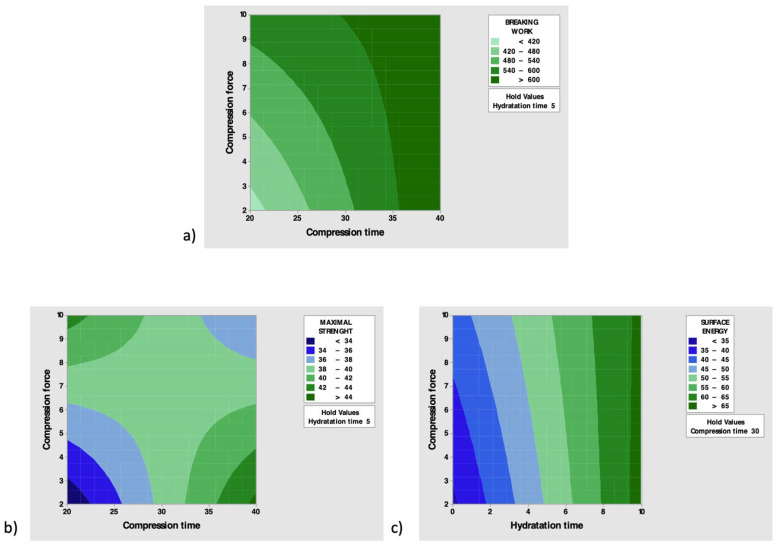
Contour plots of significate interactions for (**a**) breaking work, (**b**) maximal strength and (**c**) surface energy.

Contour plots of the most significant interactions for each response were then constructed (Figure 6).

Figure 6a shows that the greatest values of breaking work are achieved when high values of compression force are combined with the high values of compression time.

As the compression force increases and the compression time increases, the maximal strength increases (Figure 6b), while the high level of hydration time leads to the maximum surface energy, regardless of the compression force (Figure 6c).

### 4.3. Mucoadhesion Test Applied on Chitosan-Based Microparticles (CH-MLC)

Table 7 summarizes the values of surface energy (γ_s_) and cohesion work (w_c_) of the different types of tablets prepared by following the optimization setup as described above. The combination of 20 s of compression time and 10 T of compression force was applied in the production of the tablets. According to results of the Design of Experiment reported above, before each experiment, the tablets underwent the hydration phase.

The results highlighted that the presence of lactose in the ratios 20:80 and 40:60 and the presence of microparticles in the ratio 20:80 with mucin do not increase the affinity of the tablet surface towards the liquid compared to mucin alone. In fact, the surface-energy values are comparable among them. Otherwise, the presence of microparticles in a ratio 40:60 with mucin increases the wettability of the tablets (*γs* = 71 mN/m).

**Figure 7:**
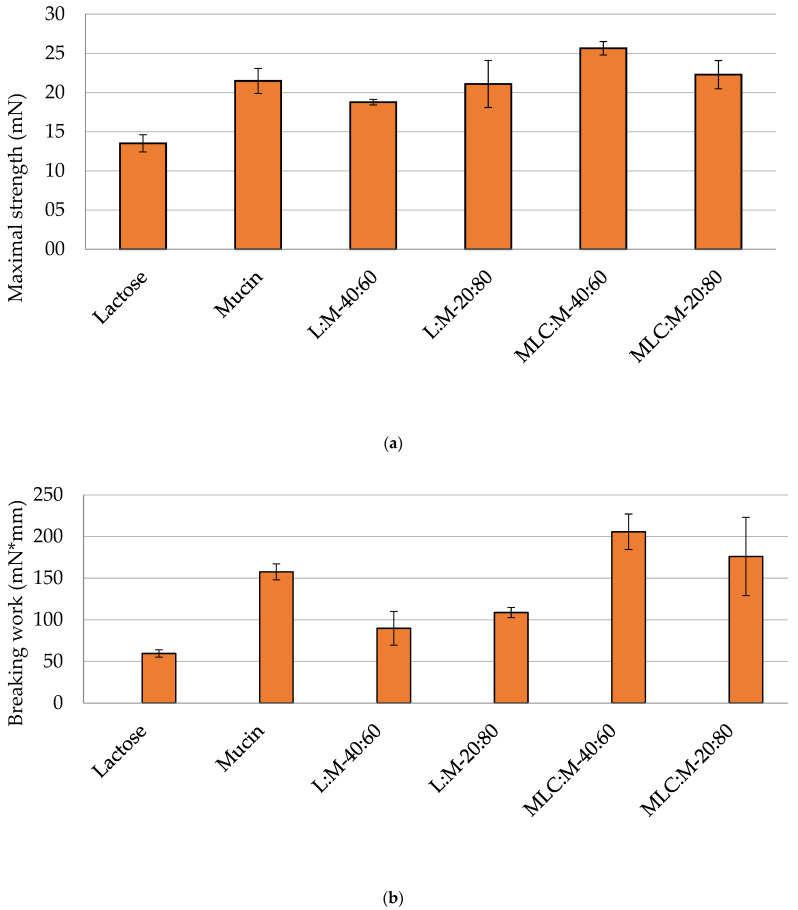
Maximal strength (**a**) and breaking work (**b**) of tablets made of lactose, mucin, lactose:mucin 40:60 *w*/*w* (L:M-40:60), lactose:mucin 20:80 *w*/*w* (L:M-20:80, CH-MLC:mucin 40:60 *w*/*w* (MLC:M-40:60) and CH-MLC:mucin 20:80 *w*/*w* (MLC:M-20:80).

The force required for the detachment of the tablet (maximal strength) from the gel (Figure 7a) is minimal for tablets containing lactose, not being a mucoadhesive molecule. This result confirmed that Caesalpina gum does not exert any force to retain non mucoadhesive molecules. In addition, the force required to cause the breakage of the mucoadhesive bonds between tablet and gel was almost the same for tablets containing chitosan microparticles as for mucin-only tablets (Figure 7a). In contrast, the mucoadhesive-bond breakage work established between the tablet components and the gel (Figure 7b) is greater when the tablets containing chitosan-based microparticles are in a 40:60 ratio (205 ± 21 mN * mm) with mucin compared to tablets made of mucin alone. This result confirms that the presence of chitosan increases the mucoadhesion of the tablets in both ratios, due to its interaction with mucin.

**Figure 8:**
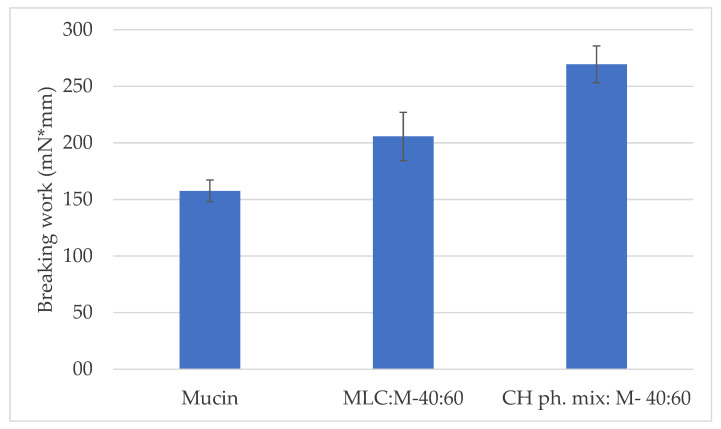
Comparison of breaking work values between tablets containing mucin, CH physical mixture (CH ph.mix) or microparticles (MLC) 40:60 with mucin (M).

To assess the behavior of free chitosan—meaning, not included in a pharmaceutical form—tablets containing the main components of the microparticles (polymer, surfactant, lipids and cryoprotectant) in a physical mixture and mucin (40:60 *w*/*w*) were analyzed. Figure 8 shows the comparison between the breaking work values obtained for the mucin tablets and the tablets containing the physical mixture (CH physical mixture:mucin) or the microparticles (CH-MLC:mucin) in the same weight ratio (40:60). The breaking work was in fact considered the most significant mucoadhesive parameter. The chitosan present in free form in the physical mixture gives the system a high mucoadhesive capacity, as the polymer is readily available to interact with the mucin. Therefore, the work required to break the mucoadhesive bond (269 ± 16 mN * mm) is greater than that required in the case of chitosan formulated within the microparticles (206 ± 21 mN * mm), and in the case of the mucin tablet (158 ± 9 mN * mm).

#### NAC Release through Artificial Mucus (AM)

**Figure 9:**
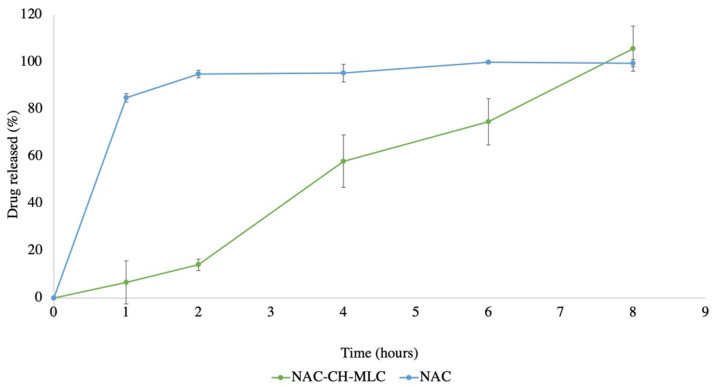
Drug-release % through artificial mucus over time of NAC-CH-MLC and pure NAC.

Figure 9 shows that the passage through the mucus of pure NAC is complete after 1 h, and that the NAC encapsulated in microparticles (NAC-CH-MLC) is completely released (105.8% ± 9.64) after 8 h.

## 5. Discussion

The mucin–chitosan interaction at the molecular level was initially studied with the turbidimetric assay. Mucoadhesive interactions between mucin and chitosan are complex, involving electrostatic interaction, as well as hydrogen bonding and hydrophobic effects. An important factor to consider is the pH, especially a strong acid pH, because of the degree of ionization of sialic acid. In a pH 4.5 solution, mucin (pKa 2.6), which presents sialic acid residues in ionized form, gives to the glycoprotein an overall negative charge; on the contrary, chitosan (pKa 6.5), at the same pH, presents a large amount (>90%) of amino groups in the ionic form. Moreover, when hydrated, the polymer chains increase their flexibility and can interpenetrate between mucin molecules, starting the process of mucoadhesion [22,23].

The turbidimetric assay proved that the maximum change in absorbance occurs when the ratio (*w*/*w*) between mucin and chitosan is 7:1 and that the molecular weight of chitosan does not affect it (*p*-value = 0.918).

From the study of the second experimental design (DoE II) it was possible to obtain the combination of compression force, compression time and optimal hydration time to test the mucoadhesive power of chitosan, both free and in microparticles. For this purpose, mucoadhesion properties of mucin tablets in contact with a gel made of sodium hyaluronate 2% were evaluated (Figure 4). Sodium hyaluronate is a mucoadhesive polymer that is capable of originating intense mucoadhesive bonds, not only thanks to the physical interpenetration of the polymer chains among those of glycoproteins (as occurs for carbomer, cellulose and PVP), but also thanks to the formation of hydrogen bonds and secondary hydrophobic interactions. Its flexibility provides it with a great resistance to the application of a tensile stress. For these reasons, it was chosen to standardize the tensile test with mucin tablets [24]. It is very important, at this early stage of setup, to use an established mucoadhesive polymer. Considering this, B01 and C01 tablets seem to be the ones that form the best mucoadhesive bonds. They represent a compromise between optimal time and compression force to form tablets with flexible particles to create both chemical and physical mucoadhesive interaction and, at the same time, to produce tablets with some strength and not too fragile. The D01 tablets were found to be the most brittle, having been compressed for less time and with less force. Furthermore, the hydration phase shows to be optimal to allow an increase in mucoadhesion activity, contrary to what was suggested by Tamburic et al. [25], who noticed that the bio-adhesion decreases with a pre-hydration phase. In fact, as the surface energy of the tablets increases, the ability to create optimal interactions in the first contact phase between the adhesive polymer and the substrate increases, and after the consolidation of the bonds, a greater force is required to be able to break them.

Once found the best combination for the tablet production, the tensile-test method was applied to tablets based on lactose (as a negative control), mucin (as a positive control), on different ratio of lactose and mucin (20:80, 40:60 *w*/*w*), different ratio CH-MLC and mucin (20:80, 40:60 *w*/*w*) and on a CH-MLC physical mixture and mucin. According to the fracture theory, the force required to cause the breakdown of the mucoadhesive bond is an indirect measure of the mucoadhesive capacity: the greater the effort required, the greater the intensity of the interaction, while the work of adhesion is the total energy involved for this separation, and it can be calculated under the area of force–distance curve. Moreover, Ponchet et al. [7] discussed a model in which the strength of a mucoadhesive bond is a function of both the interaction energy between the adhesive and the mucosa, and the viscoelastic properties of the interfacial layer formed between the two surfaces.

In this study, a different methodology approach for mucoadhesion evaluation to some previously described methods was chosen [26,27,28]. Usually, the tensile test is applied by putting in contact a tablet containing the sample to be tested for its mucoadhesive properties with a mucoadhesive medium or animal mucous tissue, or vice versa, using a mucoadhesive tablet in contact with a non-mucoadhesive gel. In the present work, a mixture of mucin and chitosan-containing microparticles was compacted into a tablet, and the chosen medium was a gel based on a non-mucoadhesive natural gum. In this way, it was possible to assess the mucoadhesive power between chitosan and mucin, firstly evaluated in solution by turbidimetric method, and then tightly bound in a non-mucoadhesive environment, which mimicked the presence of the air surface liquid (ASL) in the conductive tract of the lung. It could be seen that the strength of adhesion increased in tablets inside CH-MLC, particularly in a ratio of 40:60 (Figure 7), proving that the greater the amount of chitosan inside the tablet, the greater the mucoadhesive capacity. This is confirmed by the results in Figure 8, where free-form chitosan in the physical mixture gives the system the greatest mucoadhesive capacity, as the polymer is readily available to interact with the mucin. The first physical step of interpenetration of the polymer chains between the glycoprotein chains is more immediate, and, consequently, mucoadhesive interaction occurs more rapidly, resulting in a stronger bond. Overall, the measured mucoadhesive capacity for CH ph.mix:M (40:60) and MLC:M (40:60) tablets is higher than that measured for the mucin tablets. Thus, this type of test showed that chitosan maintains a close bond with mucin even in the presence of a fluid and even encapsulated in microparticles. Not least, it was also found that this method allows the mucoadhesive power of chitosan to be assessed even at low concentrations.

Another advantage of this new approach was not to use a solution of chitosan and mucin, where the pH, as well as the presence of other chemicals, can change the contribution of each physical interaction [12], but the use of a solid support, as mucin tablets. They made it possible to easily evaluate the surface tension, assess the cohesion work between the particles and estimate the mucoadhesive power of the polymer and/or of the pharmaceutical form containing the polymer.

In the drug delivery study, the choice of using a bioreactor was made because this system is modular, with chambers designed to be added in sequence or in parallel, thus simulating a multiple-organ system [21]. Furthermore, it is designed to be consistent with plates or transwells, thus allowing the use of standard protocols for in vitro procedures. There are two different types of diffusion-controlled system: the monolithic device and the reservoir device. NAC-CH-MLC is a monolithic system in which the drug is intimately mixed with the lipidic/polymeric matrix. Therapeutic agents released from a monolithic system are characterized by a prolonged drug release, and not to a zero-order kinetics [20], and this is confirmed by the results from the NAC release through a mucus layer. They show that the pure NAC, being a hydrophilic compound, does not suffer from any obstacle, except for the mucus viscosity, to be able to pass into the acceptor compartment. In contrast, NAC within the MLC takes much longer to be fully released, showing a more controlled profile. In fact, chitosan inside MLCs acts as a penetration enhancer: microparticles in contact with the physiological solution swell and expose the polymer chains, which interpenetrate with the glycoprotein chains of the mucin. In this way, the microparticles can adhere to the mucus and release the active precisely where it needs to act.

## 6. Conclusions

With the new approach proposed in this work, it is possible to evaluate mucoadhesive properties of chitosan-based drug delivery systems in conditions simulating the “in vivo” conditions.

In this study, the work required to cause the breakdown of the mucoadhesive bond was used as an indirect measure of the mucoadhesive capacity: the greater the effort required, the greater the intensity of the interaction. Results obtained from this work highlighted that the presence of chitosan, even if at low concentrations, strongly affects the ability of mucin tablets to form strong mucoadhesive bonds with the gel. In fact, the physical and electrostatic interaction between chitosan and mucin permitted the increase of the mucoadhesion of the overall system, requiring a greater effort to cause the detachment from the gel as compared to mucin-only tablets. In conclusion, the new proposed method makes it possible to evaluate the mucoadhesive capability of polymers, even if at low concentrations and within pharmaceutical forms. These features make this method innovative, as it may open new perspectives in the evaluation of pharmaceutical forms also in “in vivo” simulating conditions in which, usually, only a very low concentration of polymer is able to exert mucoadhesion. It is also important to consider that this method is inexpensive and easy to perform.

Finally, thanks to the use of the reactor system, it was possible to mimic the release of NAC from MLCs under dynamic conditions in vitro, demonstrating how the chitosan inside the microparticles acts as a penetration enhancer, and how the microparticles can impart a prolonged release over time.

## Figures and Tables

**Table 1:** Factors and levels of full factorial design 3^2^_._

Factors	Levels		
	−1	0	+1
MW	86,000	300,000	1,323,000
mucin: chitosan (*w*/*w*)	1:1	3:1	7:1

**Table 2:** Factors and levels of full factorial design 2^3^.

Factors	Levels	
	+1	−1
Compression time (minutes)	40	20
Compression force (tons)	10	2
Hydration time (minutes)	0	10

**Table 3:** Mucin tablets production factors.

Sample	Compression Force (Tons)	Compression Time (Seconds)
A01	10 (+1)	40 (+1)
B01	10 (+1)	20 (−1)
C01	2 (−1)	40 (+1)
D01	2 (−1)	20 (+1)

**Table 4:** Analysis of variance DoE I.

Source	Sum of Squares	Degrees of Freedom	Mean Square	*p*-Value
Molecular Weight	0.000047	2	0.000023	0.918
mucin:chitosan	0.036680	2	0.018340	0.000
2-way interactions MW mucin:chitosan	0.000777	4	0.000194	0.592
Error	0.004881	18	0.000271	
R-squared = 88.48 percent; R-squared (adjusted for df) = 83.37 percent				

**Table 5:** Surface energy (γ_s_) (±SD) and cohesion work (w_c_) (±SD) of mucin tablets hydrated and not hydrated.

Not Hydrated Tablets	γ_s_ (mN/m) (±SD)	w_c_ (mN/m) (±SD)
A01	43 ± 2	87 ± 4
B01	42 ± 0.3	84 ± 0.7
C01	30 ± 1	60 ± 2
D01	38 ± 3	76 ± 6
**Hydrated Tablets**		
A01	63 ± 0.1	127 ± 0.2
B01	69 ± 4	138 ± 8.
C01	65 ± 1.7	131 ± 4
D01	68 ± 4	137 ± 9

**Table 6:** Variance analysis DoE II.

	Sum of Squares	Degrees of Freedom	Mean Square	*p*-Value
Compression time	40.45 ^(1)^ 163955 ^(2)^ 87.78 ^(3)^	1	40.451 ^(1)^ 163955 ^(2)^ 87.78 ^(3)^	0.256 ^(1)^ 0.000 ^(2)^ 0.003 ^(3)^
Compression force	4.90 ^(1)^ 33531 ^(2)^ 91.11 ^(3)^	1	4.902 ^(1)^ 33531 ^(2)^ 91.11 ^(3)^	0.687 ^(1)^ 0.026 ^(2)^ 0.002 ^(3)^
Hydration time	85.89 ^(1)^ 82 ^(2)^ 4742.10 ^(3)^	1	85.888 ^(1)^ 82 ^(2)^ 4742.10 ^(3)^	0.105 ^(1)^ 0.905 ^(2)^ 0.000 ^(3)^
Two-way interaction				
Compression time-Compression force	530.89 ^(1)^ 48728 ^(2)^ 17.96 ^(3)^	1	530.893 ^(1)^ 48728 ^(2)^ 17.96 ^(3)^	0.001 ^(1)^ 0.009 ^(2)^ 0.129 ^(3)^
Compression time- Hydration time	270.11 ^(1)^ 13673 ^(2)^ 2.09 ^(3)^	1	270.113 ^(1)^ 13673 ^(2)^ 2.09 ^(3)^	0.008 ^(1)^ 0.136 ^(2)^ 0.592 ^(3)^
Compression force- Hydration time	3.18 ^(1)^ 20858 ^(2)^ 136.04 ^(3)^	1	3.180 ^(1)^ 20858 ^(2)^ 136.04 ^(3)^	0.745 ^(1)^ 0.071 ^(2)^ 0.000 ^(3)^
Three-way interaction				
Compression force-Compression time- Hydration time	219.45 ^(1)^ 449 ^(2)^ 46.64 ^(3)^	1	219.453 ^(1)^ 449 ^(2)^ 46.64 ^(3)^	0.014 ^(1)^ 0.780 ^(2)^ 0.020 ^(3)^
Error	465.51 ^(1)^ 88923 ^(2)^ 111.85 ^(3)^	16	29.094 ^(1)^ 5558 ^(2)^ 6.99 ^(3)^	
R-squared ^(1)^ = 71.27 percent; R-squared (adjusted for df) = 68.70 ^(1)^ percent R-squared ^(2)^ = 75.98 percent; R-squared (adjusted for df) = 65.47 ^(2)^ percent R-squared ^(3)^ = 97.86 percent; R-squared (adjusted for df) = 96.93 ^(3)^ percent				

Output: ^(1)^ maximal strength, ^(2)^ breaking work and ^(3)^ surface energy.

**Table 7:** Surface energy (γ_s_) and cohesion work (w_c_) of tablets composed of lactose mucin, lactose:mucin 40:60 *w*/*w* (L:M-40:60), lactose:mucin 20:80 *w*/*w* (L:M-20:80, CH-MLC:mucin 40:60 *w*/*w* (MLC:M-40:60), CH-MLC:mucin 20:80 *w*/*w* (MLC:M-20:80) and MLC physical mixture:M 40:60 *w*/*w* (CHph.mix:M-40:60)).

Tablets	γ_s_ (mN/m) (±SD)	w_c_ (mN/m)
Lactose	-	-
Mucin	65 ± 0.01	129 ± 0.02
L:M-40:60	64 ± 1	129 ± 1.9
L:M-20:80	63 ± 0.3	127 ± 0.5
MLC:M-40:60	71 ± 1	142 ± 3
MLC:M-20:80	64 ± 0.3	129 ± 0.8
CH ph.mix:M-40:60	70 ± 0.1	139 ± 0.2
